# Federated Networks for Distributed Analysis of Health Data

**DOI:** 10.3389/fpubh.2021.712569

**Published:** 2021-09-30

**Authors:** Harry Hallock, Serena Elizabeth Marshall, Peter A. C. 't Hoen, Jan F. Nygård, Bert Hoorne, Cameron Fox, Sharmini Alagaratnam

**Affiliations:** ^1^Healthcare Programme, Group Research and Development, DNV, Oslo, Norway; ^2^Center for Molecular and Biomolecular Informatics, Radboud Institute for Molecular Life Sciences, Radboud University Medical Center, Nijmegen, Netherlands; ^3^Department of Registry Informatics, Cancer Registry of Norway, Oslo, Norway; ^4^Industry Technology Strategy for Western Europe Health, Microsoft, Bruges, Belgium; ^5^Platform for Shaping the Future of Health and Healthcare, World Economic Forum, New York, NY, United States

**Keywords:** health data sharing, privacy, orchestration, interoperability, governance, decentralization, federated health data networks, federated learning

## Abstract

Access to health data, important for population health planning, basic and clinical research and health industry utilization, remains problematic. Legislation intended to improve access to personal data across national borders has proven to be a double-edged sword, where complexity and implications from misinterpretations have paradoxically resulted in data becoming more siloed. As a result, the potential for development of health specific AI and clinical decision support tools built on real-world data have yet to be fully realized. In this perspective, we propose federated networks as a solution to enable access to diverse data sets and tackle known and emerging health problems. The perspective draws on experience from the World Economic Forum Breaking Barriers to Health Data project, the Personal Health Train and Vantage6 infrastructures, and industry insights. We first define the concept of federated networks in a healthcare context, present the value they can bring to multiple stakeholders, and discuss their establishment, operation and implementation. Challenges of federated networks in healthcare are highlighted, as well as the resulting need for and value of an independent orchestrator for their safe, sustainable and scalable implementation.

## Introduction

Healthcare institutions generate and store health-related data from the patients in their care; in 2018 an estimated 8.41 petabytes of health data was managed by healthcare institutions (1). This data has great potential for improving diagnostic accuracy and treatment outcomes of both common and rare diseases, yet access or sharing of this data outside the host institution is often very limited (2).

## The Problem: Health Data Is Siloed

Multiple factors contribute to the siloing of health data, including unclear ownership, inadequate consent to sharing or use of data, and terms of use in data sharing/use agreements (2). Privacy concerns play a major role, where institutional and departmental interpretation of national privacy laws and the General Data Protection Regulation (GDPR) in Europe leave many clinicians and researchers unsure of the legality of sharing or providing access to patient data for primary or secondary analyses (3), and without clarity if data would be considered anonymous data in many cases.

Additionally, the custom system architectures and infrastructures, data formats, standards and cybersecurity protocols that healthcare institutions typically operate with, result in poor interoperability between different healthcare institutions. Pooling of data into centralized health databases is one approach employed to circumvent interoperability issues such as for US National Institutes of Health's Database of Genotypes and Phenotypes (4) or the European Genome-phenome Archive (5). However, concerns are growing as to the sustainability of duplicating data and the necessary storage capacity, and the increasing number of competing initiatives which in turn again fragment the data (2).

Breaking down silos of health data was the starting point of a seminar on federated analytics hosted by BigMed (6), Norway's largest precision medicine initiative. While this has long been a recognized need (7), access to relevant health data for clinical decision making and development of new treatments remains challenging (8). The implications of this are particularly relevant in precision medicine, which relies upon knowledge generated from individual patients to enable discovery for diagnosis and customization of treatment strategies, both for the current as well as future patients.

## A Solution: Federated Networks

Federated networks (FN) or data systems have been proposed as a solution to address the siloing of health data and current barriers to data sharing. While these have been differently defined in various contexts using different terms i.e., federated model (9) and federated data system (10), we propose the following definition: a FN is a series of decentralized, interconnected nodes, which allows data to be queried or otherwise analyzed by other nodes in the network without the data leaving the node it is located at. As opposed to data sharing, transfer or pooling, FNs facilitate data access or data visiting, meaning queries and algorithms can be sent to and applied on the (typically) pseudonymized data. In this paper we aim to define the hallmarks and common denominators of FNs, thus allowing for the wide range of technical solutions and architectures that fulfill these criteria to respond to the needs and requirements of their individual consortia. With that, FNs can be said to share the following common characteristics:

- Each node is semi-autonomous as they can make their own decisions on granting data access, however nodes are governed by a common framework agreed upon by all member nodes.
- FNs are supported by a common infrastructure with harmonized interoperability standards and tools.
- Each member node requires local computing capabilities to enable querying or processing to be performed locally. This is especially relevant when training AI and ML models through FNs i.e., federated learning, which may require high-performance computing.

Specifically for healthcare, Federated Health Data Networks (FHDNs) can facilitate access to sensitive health data, and have the potential to enable large cohort analysis across healthcare institutions, regional, and national borders (10, 11). Within the precision medicine paradigm and for the development of clinical decision support software in particular, FHDNs have the potential to facilitate the exchange of algorithms and queries between nodes to be executed on a set of cohort data, with the query results being returned to the requesting node, and/or algorithms being refined. The latter is termed federated learning (12), and has particular relevance in healthcare by allowing the training of a shared global algorithm on distributed sets of sensitive health data which typically does not leave their home nodes. Examples of existing clinical applications of federated learning networks include the Federated Tumor Segmentation network of 30 healthcare institutes working to improve tumor boundary detection (13), the AI4VBH (AI for value based healthcare) project, focusing on improving patient pathways in cancer, coronary artery disease, stroke, and COVID-19 using federated learning across 12 NHS trusts (UK hospitals) (14), and the Kaapana project, working through the Joint Imaging Platform across 36 German university hospitals with a focus on radiological and radiotherapeutical imaging data analysis to enable compliant and standardized approaches to imaging analysis within large-scale multi-center studies (15). A deeper look at FHDNs through the examination of the Personal Health Train and Vantage6 implementations is described below.

Multiple initiatives building on this concept have been launched, including GAIA-X, which aims to create a federated system based on common standards which connects centralized and decentralized infrastructures across industries in Europe to make data and services available (16). The proposed European Health Data Space (EHDS) which aims to promote better exchange and access to health data for primary healthcare delivery, research, and health policy (17), is one such space which GAIA-X could leverage, yet exactly how these and other related initiatives will scale and interact remains to be seen.

## Establishing a FHDN

As part of the “Breaking Barriers to Health Data” initiative (18), the World Economic Forum (WEF) published a whitepaper articulating the necessary steps to build an effective FHDN. The creation of FHDNs was found to require three core constituents to be in place; economics, governance, and technology, split between eight sequential steps (19). Notably, only two of these steps are focused on technical needs and standards i.e., structuring the data and API deployment; however this should not be underestimated, as the usability of FHDNs are limited if data and metadata interoperability (e.g., harmonization of data concepts, structures, or ontologies) is not addressed. The preceding six steps focus on relationship building i.e., trust, and policy. Alignment of incentives and recognition of available resources help to define FHDN scope, and a governance model specific to the FHDN will address common operating standards relating to inclusion criteria of data, intellectual property and responsibilities. The creation of a governance model may require substantial resources for the technical, legal, and leadership alignment within and between organizations. This should ensure patient trust, ethical use of sensitive data and trustworthiness between members before a FHDN consortium can be operationalized.

The establishment of FHDNs was recognized to offer economic return on investment in terms of diagnostic, clinical, clinical trials, and personal benefits. Although the incentivization from the economic value of these will vary between different countries, their societal value, as measurable through improved quality of life, productivity, and lifestyle of individual citizens, potentially resulting in a decreased burden on healthcare, is common to all countries.

## Operating a FHDN

**Figure 1:**
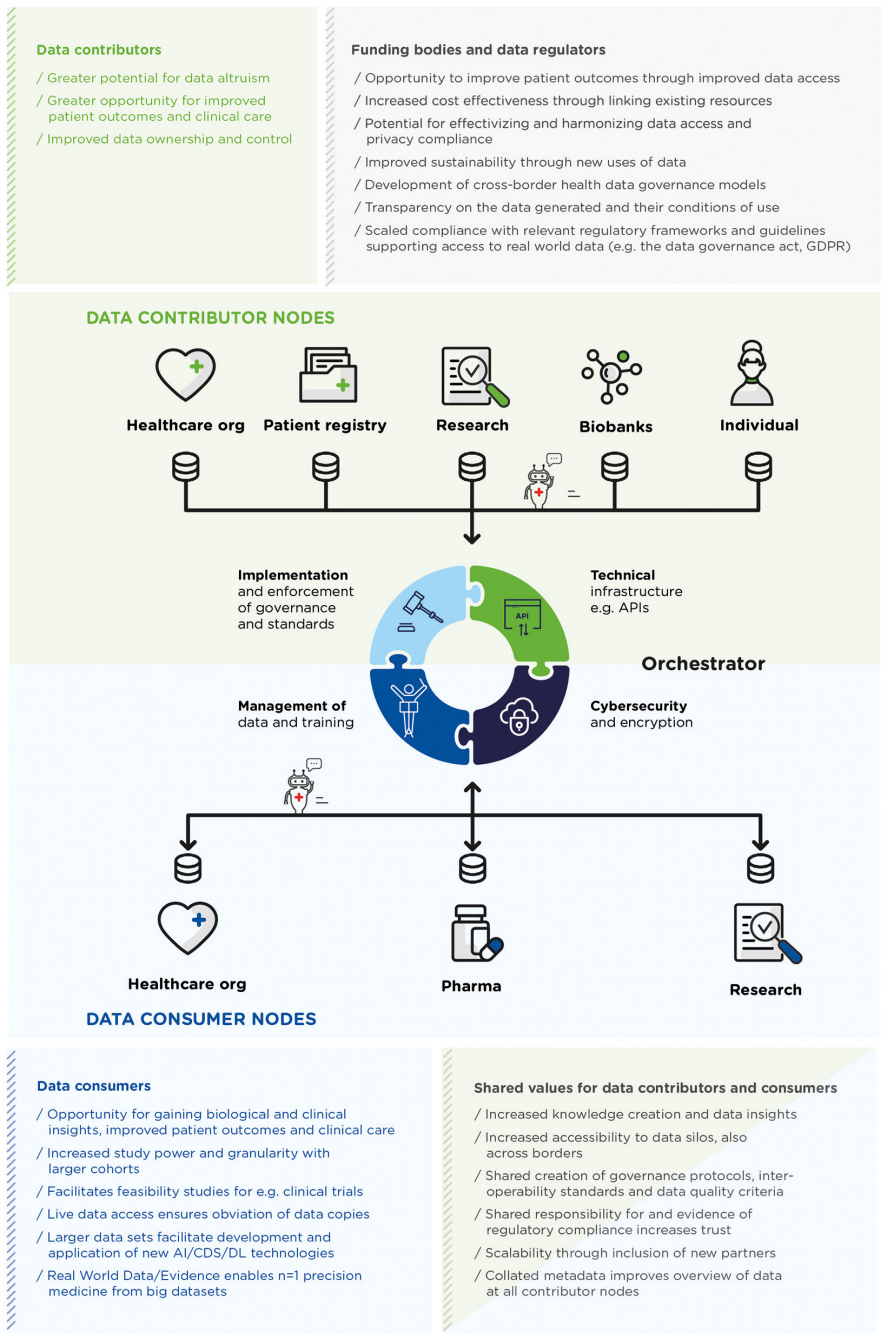
A generalized depiction of a federated health data network (FHDN), where semi-autonomous, interconnected healthcare entities act as nodes to contribute (upper green panel) and/or consume (lower blue panel) data. The infrastructure facilitates sending queries and/or algorithms (depicted here as moving robots) between nodes to visit the data stored locally, and returning, and aggregation of results. A central orchestrator can provide this infrastructure, coordinate, harmonize, and govern these activities, including in more complex set-ups, the aggregation and further distribution of results and models for their iterative improvement. The values provided to various stakeholders within a FHDN are listed by stakeholder category.

Once a FHDN has been established, the focus must switch to its operation, with its own set of requirements, specifically relating to delivering and maintaining infrastructure, cybersecurity, implementation and enforcement of governance and standards, and management of data (see Figure 1). Lightweight **infrastructure** that is compatible with potentially unreliable, slow and overwhelmed network connections (12), is a requirement for nodes to be able to exchange queries and execute algorithms, for example APIs set up when establishing the FHDN, the last step in the WEF eight-step guide (19), which must be maintained throughout the FHDN's life-cycle. Protection of sensitive difficult-to-obtain health data sets through **Cybersecurity** and privacy enhancing technologies, including network protection and robust authentication and authorization, is needed to verify nodes and potentially varying levels of access control. Encryption prior to accessing data may be needed depending on the sensitivity and anonymity of the data. Whilst in principle cybersecurity can be managed by each node separately, there is a need to mitigate the risk of differing levels of cybersecurity at each node and engender trust, which can be accomplished by an orchestrator.

Both common operating standards, for example relevant common data formats and structures, quality thresholds, biases, technical imbalances and agreed data definitions required to allow interoperability between nodes (11), and the governance framework agreed upon during the establishment of a FHDN, need to be **implemented, enforced, and monitored** across all participating nodes. This can be achieved either through autonomous adoption by the individual nodes, or through a more managed approach by incorporating use and compliance of standards and governance into the architecture of the FHDN. Additionally, during the lifetime of a FHDN, traceability, accountability and reproducibility need to be reflected in operating standards and governance which are expected to evolve to respond to new and developing needs, underlining the necessity of monitoring, adapting and refining activities to keep both standards and governance relevant and fit-for-purpose.

Depending on how the FHDN is structured, there can also be a need for active oversight and **management of potentially incomplete, unsynchronized and heterogenous data, queries, and algorithms** being exchanged within the network, and of how these queries and algorithms interact and operate with the data at the individual nodes. This includes for example the management of iterations, model compression (e.g., averaging and consensus training), and computation when the FHDN is used for federated learning, and the collation and availability of metadata about the datasets which would additionally improve their accessibility for all relevant users.

Figure 1 depicts a FHDN, the value they provide to stakeholders across the healthcare landscape and the role of the “orchestrator,” who can fulfill the responsibilities and activities detailed above.

The challenges both of establishing and sharing data within a FHDN should not be underestimated. Here we consider the role of both public and private partners contributing to the development and efficient leveraging of necessary new technologies such as cloud and machine learning for FHDNs, where industry can bring practical expertise on implementation from other sectors. Investing, co-developing and building upon low-barrier entry tools such as open-source software and standards will incentivize data sharing and the shift away from today's unsustainable paradigm of building custom solutions from scratch for each new application.

In particular, an independent body can provide expertise in aligning multiple stakeholders for a common goal, and the activities and needs pertaining to the operation of a FHDN, through the role of an **orchestrator**. Whilst orchestration activities can be distributed amongst nodes in smaller networks, the increased burden in larger and more complex networks could benefit from an orchestrator, who can deliver equally to all individual nodes of the FHDN and meet their standards, trust and needs requirements. An independent body can also fulfill a critical need for trust through a range of specific mechanisms, including standards development, risk management, accreditation, verification, validation and governance of data, algorithms, security, and quality, while developing new financial models that support both sustainable and scalable operation of FHDNs. In this way they can facilitate the sharing of publicly held data; as suggested in the proposed EU Data Governance Act (20), where a “data intermediary” could accelerate secondary use of health data.

## Example Initiatives and Implementation: Personal Health Train and Vantage6

Personal Health Train (PHT) (21) is a FHDN that allows for federation to the most granular level, that of the individual citizen. PHT uses the metaphor of trains; queries or algorithms (“trains”) travel to nodes (“stations”) that contain health data, where nodes can be not just hospital information systems or institutional databases, but also personal health records from individual citizens, as exemplified by cross-institutional patient matching (22) and identification of risk of cardiac-related hospitalizations (23) from electronic health records. Tracks define the technical specifications, standards and minimum requirements that PHT nodes and queries or algorithms should adhere to. Data remains at the nodes they were generated and are visited by queries or algorithms from data consumers i.e., healthcare providers, quality auditors, fellow citizens, or researchers. The orchestrator (“handling station”) acts as a centralized point of trust between data consumers and data contributors, and can evaluate and monitor queries and routing, and also maintain metadata and aggregate results. In the PHT architecture, copying of data is prevented and only anonymous results are returned, although it may currently be necessary to copy data into a temporary cloud-based environment for more complicated algorithms like deep learning.

PHT nodes conform with the more general FAIR Data Point (FDP) architecture (24). A FDP is an API that allows creating, storing and querying FAIR metadata about the node, and provides access to data sets within the node that should also be FAIR (25, 26), or described through semantic models that are based on ontologies in which each data element is described through a stable Uniform Resource Identifier (URI). This generalized interoperability layer makes the data at the nodes computer-readable and interpretable; an advantage of the PHT architecture over other FHDNs.

PHT started as a Dutch initiative but soon gained European and global traction through the establishment of an international GO-FAIR PHT implementation network (24). Dutch and international PHT networks consist of a wide range of public and private parties, including pharma and IT companies, health insurers, governmental and contract research organizations, hospitals, and universities, who co-develop the general architecture and implement PHT for specific use cases.

The Covid-19 pandemic accelerated the development of the PHT infrastructure. It quickly became clear that data interoperability and access represented major barriers for understanding the mechanisms of viral spread and hindered the development of new vaccines and therapeutics. The GO-FAIR Virus Outbreak Data Network (27) created a computer-readable, semantic representation of the World Health Organization (WHO) case report form and made data from Covid-19 patients available through a network of FDPs, with the first FDPs in Europe and Africa (28). This made it possible to analyze patient characteristics and the efficacy of medicines without the need to centralize data, whilst maintaining privacy for patients. A selection of clinical data projects implementing PHT have been reported in the literature (29–33).

One final example of a technical implementation of PHT is the open-source platform Vantage6 (34). Vantage6 uses a client-server model, where a researcher can pose a question and, using their preferred programming language or statistical program, send it as a query (“task”; such as a computation request) to the orchestrator (“central server”). The orchestrator oversees processing the query and handling administrative functions such as authentication and authorization. The requested query is delivered as a Docker image to the nodes. The nodes have access to their own local data. When the query has obtained a response, the result is sent via the orchestrator back to the researcher (see Figure 1).

Large-scale deployment of existing FHDNs will require the further development and deployment of existing governance frameworks to make them more relevant and scalable. In the case of PHT for example, this would involve advanced protocols for certification and auditing systems for queries or algorithms and nodes, and automated checks on security issues, potential data leaks and privacy violations.

## Implementing FHDNs More Widely: Potential Challenges and Enablers

Despite these obvious benefits, challenges during and following implementation of FHDNs can and do arise even between partners with shared ambitions, and must be addressed before FHDNs can be implemented more widely, especially across national borders. Based on parallel challenges identified in BigMed with the clinical implantation of precision medicine (35), we have grouped the challenges of implementing FHDNs into the following categories: cultural and organizational, technological, data standards, legal and regulatory, knowledge and competence, ethical and social, and financial and political. A set of potential challenges mapping to these categories, and enablers that can help overcome them are listed in Table 1. Both the panel discussion during the BigMed federated analytics seminar and further discussions between the panelists indicate that rather than technical challenges, the most difficult challenges to overcome for FHDNs are: organizational, such as resistance to transitioning from the status quo; legal and regulatory, such as compliance with GDPR and relevant requirements; financial, such as sustainable and incentivized business models; and knowledge and competence, such as training and maintaining necessary skills. Finally, the potential challenges to implementation detailed in Table 1 will affect partners in different FHDNs to varying extents, due to variations in operations and mandates, and therefore a one-sized solution will not suit all. The feasibility of addressing such challenges depends upon the overlap and sustainability of incentives, and ideals between partners.

**Table 1:** Potential challenges that can arise during and following implementation of federated health data networks, and enablers to help overcome them.

**Categories**	**Challenges**	**Potential enablers**
Cultural and organizational	• Resistance to transitioning from traditional centralized databases to FHDNs.	• Open addressing and overcoming of resistance at different levels of an organization. • Alignment along the value chain between data contributors and consumers to ensure incentives and expectations about responsible data use and ownership of results are aligned (36).
Technological	• Variability of IT infrastructure at different healthcare organizations. • Varying type and strength of security policies at different healthcare organizations.	• Integration with existing infrastructure and cybersecurity practices of healthcare organizations. • Asynchronous federated learning methods to overcome heterogeneity between computing resources at nodes and avoid bottlenecks in real-time training (37, 38).
Data standards	• Heterogenous and biased data. • Lack of harmonized standards which facilitate interoperability. This can be particularly challenging when processing data that has already been collected and structured according to different standards.	• Agreement between nodes on standards to curate and harmonize data, metadata concepts, structures and ontologies (11). • Resource prioritization for harmonization of legacy data
Legal and regulatory	• Unclear or unachievable requirements for documented compliance with legal and regulatory obligations. • Lack of clarity how the GDPR and the proposed Data Governance Act (DGA) impact FDHNs	• Agreement between partners on common, compliant governance structures of the health data and FHDNs. • Adhering to the GDPR or equivalent data protection legislation: data consent and revocation, transparency, security and privacy, and the DGA when it comes into effect.
Knowledge and competence	• Need to initiate, develop and maintain necessary competence to establish and operate FHDNs. • Insufficient education and training of researchers, clinicians and the general public about consent and personal health data.	• Advocating ease of use principles • Recognition of best practices. • Shared learnings across local and international networks. • Education and training of all stakeholders about consent. • Education and training of all stakeholders about FHDNs.
Ethical and social	• Lengthy and sometimes disjointed approval procedures with ethics committees and data protection officers, to allow others access to one's database. • Informed consent from patients. Determining preferences through dynamic consent technologies is possible within limited environments [e.g., within PHT (39)], however wide scale implementation of these has its own barriers (40).	• Standardized data access models to engender trust and maintain data protection. • Assurance that participation in a FHDN occurs within long-term ethical guiding principles.
Financial and political	• Limited clear and successful business, incentive, and reimbursement models. • Insufficient large-scale funding initiatives, such as Horizon Europe, supporting FHDNs.	• Learning from public and private initiatives for sharing of health data across borders such as 1+ Million Genomes (and beyond) (41), the European Health Data Space (17) the European Open Science Cloud (42) and GAIA-X (16).

Despite implementation challenges, FHDNs provide an innovative and sustainable solution to overcome the barriers of data sharing in healthcare. As outlined in the WEF eight-step guide, establishment of FHDNs largely involves relationship building steps such as establishing trust, aligning incentives, and identifying resources. For the successful implementation of FHDNs, a structured approach that ensures many of the challenges listed in Table 1 is sufficiently considered and addressed, is recommended. Best practices from pioneering public and private initiatives can provide approaches for solving common challenges, where an independent orchestrator can support the sustainable operation of the FHDN, especially in ensuring interoperability, cybersecurity, infrastructure, and enforcement of governance. How successfully wide-scale access to health data can be achieved will be the determining factor in development of data-driven technologies in the future, and it is hoped that papers like this one will both raise awareness of the possibilities afforded by FHDNs and ignite efforts to initiate and sustain them.

## Data Availability Statement

The original contributions presented in the study are included in the article/supplementary material, further inquiries can be directed to the corresponding author/s.

## Author Contributions

HH, SM, SA, and P'tH contributed to conception of the manuscript and wrote the first draft. JN, BH, and CF wrote sections of the manuscript. All authors contributed to manuscript revision, read, and approved the submitted version.

## Funding

The authors acknowledge the contribution of BigMed project (IKT 259055) toward the costs for designing the figures and publication fees. PACtH's activities relevant to this work are supported by a ZON-MW grant to the FAIR Genomes (project no. 846003201) and a NWO large scale research infrastructure grant to the Netherlands X-omics Initiative (grant no. 184.034.019).

## Conflict of Interest

The authors declare that the research was conducted in the absence of any commercial or financial relationships that could be construed as a potential conflict of interest.

## Publisher's Note

All claims expressed in this article are solely those of the authors and do not necessarily represent those of their affiliated organizations, or those of the publisher, the editors and the reviewers. Any product that may be evaluated in this article, or claim that may be made by its manufacturer, is not guaranteed or endorsed by the publisher.

## References

[1] DELL Technologies. Edge and IoT solutions - A new vision for Healthcare and Life Sciences. (2019). Available online at: https://www.delltechnologies.com/en-us/collaterals/unauth/infographic/solutions/dell-technologies-edge-iot-healthcare-infographic.pdf (accessed March 29, 2021).

[2] Powell K. The broken promise that undermines human genome research. Nature. (2021) 590:198–201. 10.1038/d41586-021-00331-5 33568833

[3] Peloquin D DiMaio M Bierer B Barnes M. Disruptive and avoidable: GDPR challenges to secondary research uses of data. Eur J Hum Genet. (2020) 28:697–705. 10.1038/s41431-020-0596-x 32123329 PMC7411058

[4] NIH's Database of Genotypes Phenotypes (dbGaP). Available online at: https://www.ncbi.nlm.nih.gov/gap/ (accessed March 29, 2021).

[5] The European Genome-phenome Archive (EGA). Available online at: https://ega-archive.org/ (accessed March 29, 2021).

[6] BigMed. Federated analytics of health data. (2020). Available online at: https://bigmed.no/projects/federated-analytics (accessed May 3, 2021).

[7] Miller AR Tucker C. Health information exchange, system size and information silos. J Health Econ. (2014) 33:28–42. 10.1016/j.jhealeco.2013.10.004 24246484

[8] Khosla S White R Medina J Ouwens M Emmas C Koder T. Real world evidence (RWE) – a disruptive innovation or the quiet evolution of medical evidence generation? F1000Res. (2018) 7:13585. 10.12688/f1000research.13585.1 30026923 PMC6039945

[9] Weber GM Murphy SN McMurry AJ MacFadden D Nigrin DJ Churchill S. The shared health research information network (SHRINE): a prototype federated query tool for clinical data repositories. J Am Med Informatics Assoc. (2009) 16:624–30. 10.1197/jamia.M3191 19567788 PMC2744712

[10] World economic Forum. Federated Data Systems: Balancing Innovation and Trust in the Use of Sensitive Data. (2019). Available online at: http://www3.weforum.org/docs/WEF_Federated_Data_Systems_2019.pdf (accessed August 20, 2021).

[11] Rieke N Hancox J Li W Milletarì F Roth HR Albarqouni S. The future of digital health with federated learning. npj Digit Med. (2020) 3:119. 10.1038/s41746-020-00323-1 33015372 PMC7490367

[12] Xu J Glicksberg BS Su C Walker P Bian J Wang F. Federated Learning for Healthcare Informatics. J Healthc Informatics Res. (2021) 5:1–19. 10.1007/s41666-020-00082-4 33204939 PMC7659898

[13] Perelman school of Medicine. The Federated Tumor Segmentation (FeTS) initiative. Available online at: https://www.med.upenn.edu/cbica/fets/ (accessed August 20, 2021).

[14] AI4VBH - AI Centre for Value Based Healthcare. Available online at: https://www.aicentre.co.uk/projects (accessed July 2, 2021).

[15] Scherer J Nolden M Kleesiek J Metzger J Kades K Schneider V. Joint imaging platform for federated clinical data analytics. JCO Clin Cancer Inform. (2020) 4:1027–38. 10.1200/CCI.20.00045 33166197 PMC7713526

[16] Federal Ministry for Economic Affairs and Energy. GAIA-X: Policy Rules and Architecture of Standards. (2020). Available online at: https://www.data-infrastructure.eu/GAIAX/Redaktion/EN/Publications/gaia-x-policy-rules-and-architecture-of-standards.pdf?__blob=publicationFile&v=5%0A10%09EuropeanCommission. European Commissio (accessed April 8, 2021).

[17] European Commission: European Health Data Space. (2019). Available online at: https://ec.europa.eu/health/ehealth/dataspace_en (accessed April 8, 2021).

[18] World Economic Forum. Breaking barriers to health data project. Available online at: https://www.weforum.org/projects/breaking-barriers-to-health-data-project (accessed May 3, 2021).

[19] World Economic Forum. Sharing Sensitive Health Data in a Federated Data Consortium Model: An Eight-Step Guide. Geneva (2020). Available online at: https://www.weforum.org/reports/sharing-sensitive-health-data-in-a-federated-data-consortium-model-an-eight-step-guide (accessed August 20, 2021).

[20] European Commission. Proposal for a regulation of the European Parliament and of the council on European data governance (Data Governance Act). Brussels (2020). Available online at: https://eur-lex.europa.eu/legal-content/EN/TXT/HTML/?uri=CELEX:52020PC0767&from=EN (accessed August 20, 2021).

[21] The Personal Health Train Network. Available online at: www.personalhealthtrain.nl (accessed March 29, 2021).

[22] Lee J Sun J Wang F Wang S Jun C-H Jiang X. Privacy-preserving patient similarity learning in a federated environment: development and analysis. JMIR Med Inform. (2018) 6:e20. 10.2196/medinform.7744 29653917 PMC5924379

[23] Brisimi TS Chen R Mela T Olshevsky A Paschalidis IC Shi W. Federated learning of predictive models from federated Electronic Health Records. Int J Med Inform. (2018) 112:59–67. 10.1016/j.ijmedinf.2018.01.007 29500022 PMC5836813

[24] Fair Data Point. Available online at: https://www.fairdatapoint.org/ (accessed March 29, 2021).

[25] Wilkinson MD Dumontier M Aalbersberg IjJ Appleton G Axton M Baak A. The FAIR Guiding Principles for scientific data management and stewardship. Sci Data. (2016) 3:160018. 10.1038/sdata.2016.18 26978244 PMC4792175

[26] Wilkinson MD Sansone S-A Schultes E Doorn P Bonino da Silva Santos LO Dumontier M. A design framework and exemplar metrics for FAIRness. Sci Data. (2018) 5:180118. 10.1038/sdata.2018.118 29944145 PMC6018520

[27] GO FAIR Virus Outbreak Data Network (VODAN). (2020). Available online at: https://www.go-fair.org/implementation-networks/overview/vodan/ (accessed July 2, 2021).

[28] van Reisen M Oladipo F Stokmans M Mpezamihgo M Folorunso S Schultes E. Design of a FAIR digital data health infrastructure in Africa for COVID-19 reporting and research. Adv Genet. (2021) 2:e10050. 10.1002/ggn2.10050 34514430 PMC8420285

[29] The Personal Health Train: use cases. Available online at: https://pht.health-ri.nl/use-cases (accessed March 29, 2021).

[30] Sun C Ippel L van Soest J Wouters B Malic A Adekunle O. A privacy-preserving infrastructure for analyzing personal health data in a vertically partitioned scenario. Stud Health Technol Inform. (2019) 264:373–7. 10.3233/SHTI190246 31437948

[31] Geleijnse G Chiang RC-J Sieswerda M Schuurman M Lee KC van Soest J. Prognostic factors analysis for oral cavity cancer survival in the Netherlands and Taiwan using a privacy-preserving federated infrastructure. Sci Rep. (2020) 10:20526. 10.1038/s41598-020-77476-2 33239719 PMC7688977

[32] Deist TM Dankers FJWM Ojha P Scott Marshall M Janssen T Faivre-Finn C. Distributed learning on 20 000+ lung cancer patients – The Personal Health Train. Radiother Oncol. (*2*020) 144:189–200. 10.1016/j.radonc.2019.11.019 31911366

[33] Beyan O Choudhury A van Soest J Kohlbacher O Zimmermann L Stenzhorn H. Distributed analytics on sensitive medical data: the personal health train. Data Intell. (2020) 2:96–107. 10.1162/dint_a_00032

[34] Vantage6: priVAcy preserviNg federaTed leArninG infrastructurE for Secure Insight eXchange. Available online at: https://vantage6.ai (accessed March 29, 2021). PMC8075508 33936462

[35] Binz Vallevik Vibeke Zaka Alia Ray-Sannerud Bobbie. Reflections on the clinical implementation of precision medicine – Experiences from BigMed, a Norwegian ICT Lighthouse project. (2021). Available online at: https://bigmed.no/assets/bigmed_reflections-on-the_2021_v1.0.pdf (accessed August 20, 2021).

[36] Devriendt T Borry P Shabani M. Factors that influence data sharing through data sharing platforms: a qualitative study on the views and experiences of cohort holders and platform developers. PLoS ONE. (2021) 16:e0254202. 10.1371/journal.pone.0254202 34214146 PMC8253381

[37] Chen Z Liao W Hua K Lu C Yu W. Towards asynchronous federated learning for heterogeneous edge-powered internet of things. Digit Commun Netw. (2021) 7:317–26. 10.1016/j.dcan.2021.04.001

[38] Liu Y Qu Y Xu C Hao Z Gu B. Blockchain-enabled asynchronous federated learning in edge computing. Sensors. (2021) 21:3335. 10.3390/s21103335 34064942 PMC8151195

[39] GO FAIR Personal locker consent demo. (2018). Available online at: https://www.youtube.com/watch?v=xREv60t1Na0&feature=youtu.be (accessed March 29, 2021).

[40] DNV. Dynamic consent in clinical genetics: implementation barriers. Oslo (2021). Available online at: https://www.dnv.com/research/precision-medicine/dynamic-consent-whitepaper.html (accessed August 20, 2021).

[41] Beyond 1 Million Genomes (B1MG). (2020). Available online at: https://b1mg-project.eu/ (accessed March 29, 2021).

[42] European Open Science Cloud. (2018). Available online at: https://eosc-portal.eu/ (accessed March 29, 2021).

